# Diethyl {(4-methoxy­phen­yl)[5-(4-nitro­phen­yl)-1,3,4-thia­diazol-2-ylamino]meth­yl}phospho­nate

**DOI:** 10.1107/S1600536808017364

**Published:** 2008-07-05

**Authors:** Li-He Yin, Rong Wan, Feng Han, Bin Wang, Jin-Tang Wang

**Affiliations:** a Department of Applied Chemistry, College of Science, Nanjing University of Technology, No. 5 Xinmofan Road, Nanjing 210009, People’s Republic of China

## Abstract

The title compound, C_20_H_23_N_4_O_6_PS, was synthesized by the reaction of *N*-(4-methoxy­benzyl­idene)-5-(4-nitro­phen­yl)-1,3,4-thia­diazol-2-amine and diethyl phosphite. The thia­diazole and nitro-substituted phenyl rings in the mol­ecule are approximately coplanar, the dihedral angle being 5.3 (2)°. The dihedral angle formed by the mean plane through all non-H atoms of both the thia­diazole and the nitro-substituted phenyl ring with the plane of the meth­oxy-substituted phenyl ring is 48.9 (2)°. In the crystal structure, mol­ecules form centrosymmetric dimers as a result of N—H⋯O bonds involving amine H and phosphine oxide O atoms.

## Related literature

For related literature, see: Nakagawa *et al.* (1996 ▶); Wang *et al.* (1999 ▶).
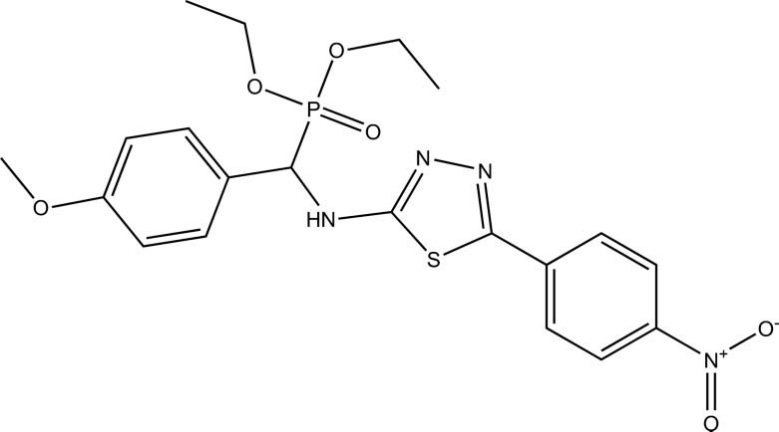


## Experimental

### 

#### Crystal data

C_20_H_23_N_4_O_6_PS
*M* *_r_* = 478.45
Monoclinic, 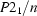

*a* = 11.481 (2) Å
*b* = 19.426 (4) Å
*c* = 11.960 (2) Å
β = 117.08 (3)°
*V* = 2375.0 (10) Å^3^
*Z* = 4
Mo *K*α radiation
μ = 0.25 mm^−1^
*T* = 298 (2) K
0.30 × 0.20 × 0.10 mm

#### Data collection

Enraf–Nonius CAD-4 diffractometer
Absorption correction: ψ scan (North *et al.*, 1968 ▶) *T*_min_ = 0.930, *T*_max_ = 0.976
4497 measured reflections
4276 independent reflections
2330 reflections with *I* > 2σ(*I*)
*R*_int_ = 0.064
3 standard reflections every 200 reflections intensity decay: none

#### Refinement

*R*[*F* ^2^ > 2σ(*F* ^2^)] = 0.068
*wR*(*F* ^2^) = 0.146
*S* = 1.03
4276 reflections
289 parameters
H-atom parameters constrained
Δρ_max_ = 0.30 e Å^−3^
Δρ_min_ = −0.21 e Å^−3^

### 

Data collection: *CAD-4 Software* (Enraf–Nonius,1989 ▶); cell refinement: *CAD-4 Software*; data reduction: *XCAD4* (Harms & Wocadlo,1995 ▶); program(s) used to solve structure: *SHELXS97* (Sheldrick, 2008 ▶); program(s) used to refine structure: *SHELXL97* (Sheldrick, 2008 ▶); molecular graphics: *SHELXTL* (Sheldrick, 2008 ▶); software used to prepare material for publication: *SHELXL97*.

## Supplementary Material

Crystal structure: contains datablocks global, I. DOI: 10.1107/S1600536808017364/ya2071sup1.cif

Structure factors: contains datablocks I. DOI: 10.1107/S1600536808017364/ya2071Isup2.hkl

Additional supplementary materials:  crystallographic information; 3D view; checkCIF report

## Figures and Tables

**Table 1:** Hydrogen-bond geometry (Å, °)

*D*—H⋯*A*	*D*—H	H⋯*A*	*D*⋯*A*	*D*—H⋯*A*
N1—H1*A*⋯O5^i^	0.86	1.94	2.782 (6)	165

Symmetry code: (i) 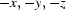.

**Fig. 1.**
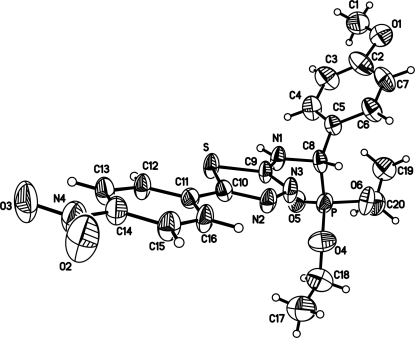
Molecular structure of (I). Displacement ellipsoids are drawn at the 30% probability level. H atoms are shown as small circles of arbitrary radius.

## supplementary crystallographic information

### Comment

1,3,4-Thiadiazole derivatives represent an interesting class of biologically important compounds, which often exhibit insecticidal, fungicidal and other biological activities (Nakagawa *et al.*, 1996; Wang *et al.*, 1999).

We report here the X-ray structure of the title compound,(I)(Fig. 1). The thiadiazole and nitro-substituted phenyl rings in the molecule of (I) are approximately coplanar: the dihedral angle between the C11—C16 and S/C10/N2/N3/C9 planes being 5.3 (2)°, the maximum deviation from the mean plane N1/C9/C10/N2/N3/S/C11/C12/C13/C14/C15/C16 does not exceed 0.11 Å. The dihedral angle formed by the latter plane with the plane of the methoxy- substituted phenyl ring C2—C7 is equal to 48.9 (2) °.

In the crystal structure, molecules of (I) form centrosymmetric dimers due to N—H···O bonds involving amine hydrogen and phosphineoxide oxygen [N1···O5^i^ 2.782 (6) Å; symmetry code (i): -*x*, -*y*, -*z*].

### Experimental

*N*-(4-methoxybenzylidene)-5-(4-nitrophenyl)-1,3,4-thiadiazol-2-amine (2 mmol) and diethyl phosphite (5 mmol) were mixed in a 25 ml flask (without any solvent),and kept in the oil bath at 90°C for 6 h. After cooling, the crude product (I) precipitated and was filtered. Pure compound (I) was obtained by recrystallization from ethanol(20 ml). Crystals of (I) suitable for X-ray diffraction were obtained by slow evaporation of an acetone solution.

### Refinement

All H atoms were positioned geometrically, with N—H=0.86 Å and C—H=0.98, 0.97, 0.96 and 0.93 Å for methine, methylene, methyl and aromatic H atoms, respectively, and constrained to ride on their parent atoms, with *U*_iso_(H)=*xU*_eq_(C, N), where *x*=1.5 for methyl H atoms and *x*=1.2 for all other H atoms.

### Crystal data

**Table:**

C_20_H_23_N_4_O_6_PS	*F*(000) = 1000
*M**_r_* = 478.45	*D*_x_ = 1.338 Mg m^−^^3^
Monoclinic, *P*2_1_/*n*	Melting point: 476 K
Hall symbol: -P 2yn	Mo *K*α radiation, λ = 0.71073 Å
*a* = 11.481 (2) Å	Cell parameters from 25 reflections
*b* = 19.426 (4) Å	θ = 9–13°
*c* = 11.960 (2) Å	µ = 0.25 mm^−^^1^
β = 117.08 (3)°	*T* = 298 K
*V* = 2375.0 (10) Å^3^	Block, light yellow
*Z* = 4	0.30 × 0.20 × 0.10 mm

### Data collection

**Table:**

Enraf–Nonius CAD-4 diffractometer	2330 reflections with *I* > 2σ(*I*)
Radiation source: fine-focus sealed tube	*R*_int_ = 0.064
graphite	θ_max_ = 25.2°, θ_min_ = 2.0°
ω/2θ scans	*h* = −13→12
Absorption correction: ψ scan (North *et al.*, 1968)	*k* = 0→23
*T*_min_ = 0.930, *T*_max_ = 0.976	*l* = 0→14
4497 measured reflections	3 standard reflections every 200 reflections
4276 independent reflections	intensity decay: none

### Refinement

**Table:**

Refinement on *F*^2^	Primary atom site location: structure-invariant direct methods
Least-squares matrix: full	Secondary atom site location: difference Fourier map
*R*[*F*^2^ > 2σ(*F*^2^)] = 0.068	Hydrogen site location: inferred from neighbouring sites
*wR*(*F*^2^) = 0.146	H-atom parameters constrained
*S* = 1.03	*w* = 1/[σ^2^(*F*_o_^2^) + (0.03*P*)^2^ + 3.2*P*] where *P* = (*F*_o_^2^ + 2*F*_c_^2^)/3
4276 reflections	(Δ/σ)_max_ < 0.001
289 parameters	Δρ_max_ = 0.30 e Å^−^^3^
0 restraints	Δρ_min_ = −0.21 e Å^−^^3^

### Special details

**Table:**

Geometry. All e.s.d.'s (except the e.s.d. in the dihedral angle between two l.s. planes) are estimated using the full covariance matrix. The cell e.s.d.'s are taken into account individually in the estimation of e.s.d.'s in distances, angles and torsion angles; correlations between e.s.d.'s in cell parameters are only used when they are defined by crystal symmetry. An approximate (isotropic) treatment of cell e.s.d.'s is used for estimating e.s.d.'s involving l.s. planes.
Refinement. Refinement of *F*^2^ against ALL reflections. The weighted *R*-factor *wR* and goodness of fit *S* are based on *F*^2^, conventional *R*-factors *R* are based on *F*, with *F* set to zero for negative *F*^2^. The threshold expression of *F*^2^ > σ(*F*^2^) is used only for calculating *R*-factors(gt) *etc*. and is not relevant to the choice of reflections for refinement. *R*-factors based on *F*^2^ are statistically about twice as large as those based on *F*, and *R*- factors based on ALL data will be even larger.

### Fractional atomic coordinates and isotropic or equivalent isotropic displacement parameters (Å^2^)

**Table:**

	*x*	*y*	*z*	*U*_iso_*/*U*_eq_	
S	0.11752 (9)	0.17107 (5)	−0.10907 (11)	0.0688 (4)	
P	0.23008 (11)	−0.02508 (6)	0.20206 (12)	0.0738 (4)	
N1	0.1765 (3)	0.04373 (17)	−0.0120 (3)	0.0700 (10)	
H1A	0.0966	0.0342	−0.0631	0.084*	
O1	0.2443 (4)	−0.2713 (2)	−0.1562 (4)	0.1097 (12)	
C1	0.1296 (7)	−0.2920 (3)	−0.2318 (6)	0.113 (2)	
H1B	0.1350	−0.3368	−0.2631	0.185*	
H1C	0.0906	−0.2603	−0.3007	0.185*	
H1D	0.0769	−0.2945	−0.1885	0.185*	
N2	0.3560 (3)	0.19583 (16)	0.0431 (3)	0.0639 (9)	
O2	0.3525 (4)	0.53251 (19)	−0.1632 (4)	0.154 (2)	
C2	0.2280 (7)	−0.2053 (3)	−0.1134 (7)	0.108 (2)	
N3	0.3379 (3)	0.12947 (16)	0.0636 (3)	0.0699 (10)	
O3	0.1531 (4)	0.52326 (17)	−0.2953 (4)	0.1379 (17)	
C3	0.1235 (6)	−0.1701 (3)	−0.1297 (6)	0.1072 (19)	
H3B	0.0416	−0.1890	−0.1795	0.129*	
N4	0.2539 (5)	0.4987 (2)	−0.2075 (4)	0.0979 (14)	
C4	0.1299 (4)	−0.1052 (2)	−0.0755 (5)	0.0913 (16)	
H4A	0.0530	−0.0821	−0.0911	0.110*	
O4	0.2896 (4)	0.04106 (19)	0.2851 (4)	0.1017 (13)	
O5	0.0918 (3)	−0.03805 (17)	0.1673 (3)	0.0877 (10)	
C5	0.2466 (4)	−0.0758 (2)	−0.0010 (4)	0.0624 (11)	
O6	0.3218 (3)	−0.0846 (2)	0.2736 (3)	0.1005 (12)	
C6	0.3601 (5)	−0.1129 (3)	0.0189 (5)	0.0917 (16)	
H6A	0.4426	−0.0944	0.0683	0.110*	
C7	0.3476 (6)	−0.1768 (3)	−0.0357 (6)	0.0997 (17)	
H7A	0.4229	−0.2017	−0.0194	0.120*	
C8	0.2618 (4)	−0.0111 (2)	0.0683 (4)	0.0660 (12)	
H8A	0.3527	0.0042	0.1000	0.079*	
C9	0.2198 (3)	0.1078 (2)	−0.0078 (4)	0.0581 (10)	
C10	0.2513 (3)	0.22694 (18)	−0.0425 (4)	0.0560 (10)	
C11	0.2495 (3)	0.29723 (19)	−0.0822 (3)	0.0553 (10)	
C12	0.1363 (3)	0.32734 (19)	−0.1726 (4)	0.0656 (12)	
H12A	0.0584	0.3026	−0.2044	0.079*	
C13	0.1361 (4)	0.3941 (2)	−0.2172 (4)	0.0795 (14)	
H13A	0.0599	0.4130	−0.2800	0.095*	
C14	0.2518 (4)	0.4315 (2)	−0.1658 (4)	0.0774 (13)	
C15	0.3662 (4)	0.4028 (2)	−0.0697 (4)	0.0732 (13)	
H15A	0.4426	0.4287	−0.0333	0.088*	
C16	0.3654 (3)	0.3367 (2)	−0.0296 (4)	0.0698 (12)	
H16A	0.4417	0.3177	0.0328	0.084*	
C17	0.2907 (7)	0.1293 (4)	0.4169 (7)	0.118 (3)	
H17A	0.2690	0.1414	0.4830	0.177*	
H17B	0.2346	0.1539	0.3418	0.177*	
H17C	0.3802	0.1413	0.4413	0.177*	
C18	0.2731 (7)	0.0561 (3)	0.3935 (7)	0.124 (2)	
H18A	0.3368	0.0309	0.4653	0.151*	
H18B	0.1863	0.0425	0.3798	0.151*	
C19	0.2659 (6)	−0.2016 (3)	0.2448 (6)	0.121 (2)	
H19A	0.2385	−0.2397	0.2783	0.182*	
H19B	0.3481	−0.2123	0.2461	0.182*	
H19C	0.2014	−0.1930	0.1600	0.182*	
C20	0.2802 (6)	−0.1418 (3)	0.3193 (6)	0.1095 (18)	
H20A	0.1973	−0.1313	0.3184	0.131*	
H20B	0.3437	−0.1510	0.4055	0.131*	

### Atomic displacement parameters (Å^2^)

**Table:**

	*U*^11^	*U*^22^	*U*^33^	*U*^12^	*U*^13^	*U*^23^
S	0.0357 (5)	0.0488 (6)	0.0856 (8)	−0.0028 (5)	−0.0039 (5)	0.0022 (6)
P	0.0516 (7)	0.0748 (8)	0.0725 (8)	0.0008 (6)	0.0087 (6)	−0.0038 (7)
N1	0.0389 (18)	0.057 (2)	0.085 (2)	−0.0010 (16)	0.0027 (17)	0.0111 (18)
O1	0.118 (3)	0.102 (3)	0.107 (3)	0.011 (3)	0.094 (3)	−0.013 (2)
C1	0.119 (5)	0.123 (6)	0.110 (5)	0.008 (5)	0.068 (5)	0.014 (4)
N2	0.0360 (17)	0.051 (2)	0.073 (2)	−0.0047 (15)	−0.0024 (15)	0.0008 (17)
O2	0.135 (4)	0.066 (2)	0.163 (4)	−0.040 (2)	−0.018 (3)	0.012 (2)
C2	0.137 (6)	0.048 (3)	0.168 (6)	−0.007 (3)	0.095 (5)	−0.018 (3)
N3	0.0392 (18)	0.050 (2)	0.087 (2)	0.0054 (15)	−0.0002 (17)	0.0068 (18)
O3	0.130 (3)	0.059 (2)	0.148 (4)	0.018 (2)	−0.003 (3)	0.015 (2)
C3	0.100 (4)	0.073 (4)	0.150 (5)	−0.031 (3)	0.058 (4)	−0.039 (4)
N4	0.091 (3)	0.057 (3)	0.096 (3)	−0.002 (2)	0.001 (3)	0.001 (2)
C4	0.066 (3)	0.058 (3)	0.124 (4)	−0.014 (2)	0.020 (3)	−0.015 (3)
O4	0.117 (3)	0.097 (3)	0.113 (3)	−0.037 (2)	0.036 (3)	−0.049 (2)
O5	0.0603 (19)	0.104 (2)	0.091 (2)	0.0058 (17)	0.0280 (17)	0.0085 (19)
C5	0.055 (2)	0.050 (2)	0.082 (3)	−0.001 (2)	0.031 (2)	0.000 (2)
O6	0.068 (2)	0.101 (3)	0.104 (3)	0.019 (2)	0.0099 (19)	0.043 (2)
C6	0.073 (3)	0.080 (4)	0.136 (5)	0.002 (3)	0.060 (3)	−0.008 (3)
C7	0.116 (5)	0.062 (3)	0.151 (5)	0.024 (3)	0.086 (4)	0.017 (3)
C8	0.036 (2)	0.057 (3)	0.096 (3)	0.0057 (18)	0.023 (2)	0.006 (2)
C9	0.032 (2)	0.051 (2)	0.072 (3)	0.0090 (17)	0.0069 (18)	0.006 (2)
C10	0.0327 (19)	0.042 (2)	0.072 (3)	−0.0039 (16)	0.0058 (18)	−0.0128 (19)
C11	0.040 (2)	0.052 (2)	0.055 (2)	0.0013 (17)	0.0056 (17)	−0.0093 (19)
C12	0.041 (2)	0.041 (2)	0.076 (3)	−0.0024 (17)	−0.0062 (19)	0.001 (2)
C13	0.055 (3)	0.046 (2)	0.089 (3)	−0.002 (2)	−0.009 (2)	0.012 (2)
C14	0.063 (3)	0.052 (3)	0.084 (3)	0.000 (2)	0.006 (2)	−0.002 (2)
C15	0.046 (2)	0.065 (3)	0.085 (3)	−0.009 (2)	0.009 (2)	0.003 (2)
C16	0.039 (2)	0.051 (2)	0.080 (3)	0.0010 (18)	−0.0073 (19)	0.004 (2)
C17	0.129 (7)	0.116 (7)	0.109 (6)	0.002 (6)	−0.037 (5)	−0.029 (6)
C18	0.136 (6)	0.108 (5)	0.127 (6)	−0.003 (4)	0.071 (5)	−0.018 (4)
C19	0.124 (5)	0.111 (5)	0.133 (5)	−0.007 (4)	0.063 (4)	−0.004 (4)
C20	0.106 (4)	0.113 (5)	0.110 (5)	0.010 (4)	0.050 (4)	0.025 (4)

### Geometric parameters (Å, °)

**Table:**

S—C10	1.749 (3)	O6—C20	1.415 (6)
S—C9	1.750 (4)	C6—C7	1.381 (7)
P—O5	1.468 (3)	C6—H6A	0.9300
P—O6	1.535 (3)	C7—H7A	0.9300
P—O4	1.577 (3)	C8—H8A	0.9800
P—C8	1.816 (5)	C10—C11	1.442 (5)
N1—C9	1.332 (5)	C11—C12	1.386 (5)
N1—C8	1.469 (5)	C11—C16	1.411 (5)
N1—H1A	0.8600	C12—C13	1.402 (5)
O1—C1	1.276 (6)	C12—H12A	0.9300
O1—C2	1.423 (6)	C13—C14	1.387 (5)
C1—H1B	0.9600	C13—H13A	0.9300
C1—H1C	0.9600	C14—C15	1.408 (5)
C1—H1D	0.9600	C15—C16	1.373 (5)
N2—C10	1.318 (4)	C15—H15A	0.9300
N2—N3	1.346 (4)	C16—H16A	0.9300
O2—N4	1.203 (5)	C17—C18	1.446 (8)
C2—C3	1.316 (7)	C17—H17A	0.9600
C2—C7	1.376 (7)	C17—H17B	0.9600
N3—C9	1.301 (4)	C17—H17C	0.9600
O3—N4	1.251 (5)	C18—H18A	0.9700
C3—C4	1.404 (6)	C18—H18B	0.9700
C3—H3B	0.9300	C19—C20	1.426 (7)
N4—C14	1.402 (6)	C19—H19A	0.9600
C4—C5	1.352 (5)	C19—H19B	0.9600
C4—H4A	0.9300	C19—H19C	0.9600
O4—C18	1.423 (7)	C20—H20A	0.9700
C5—C6	1.412 (6)	C20—H20B	0.9700
C5—C8	1.471 (5)		
			
C10—S—C9	87.00 (18)	N3—C9—N1	125.9 (3)
O5—P—O6	114.1 (2)	N3—C9—S	113.4 (3)
O5—P—O4	114.9 (2)	N1—C9—S	120.6 (3)
O6—P—O4	105.8 (2)	N2—C10—C11	124.1 (3)
O5—P—C8	113.63 (19)	N2—C10—S	111.7 (3)
O6—P—C8	104.0 (2)	C11—C10—S	124.0 (3)
O4—P—C8	103.3 (2)	C12—C11—C16	118.6 (4)
C9—N1—C8	121.8 (3)	C12—C11—C10	121.6 (3)
C9—N1—H1A	119.1	C16—C11—C10	119.8 (3)
C8—N1—H1A	119.1	C11—C12—C13	121.7 (4)
C1—O1—C2	106.1 (5)	C11—C12—H12A	119.1
O1—C1—H1B	109.5	C13—C12—H12A	119.1
O1—C1—H1C	109.5	C14—C13—C12	118.6 (4)
H1B—C1—H1C	109.5	C14—C13—H13A	120.7
O1—C1—H1D	109.5	C12—C13—H13A	120.7
H1B—C1—H1D	109.5	C13—C14—N4	119.8 (4)
H1C—C1—H1D	109.5	C13—C14—C15	120.3 (4)
C10—N2—N3	114.8 (3)	N4—C14—C15	119.8 (4)
C3—C2—C7	116.9 (5)	C16—C15—C14	120.2 (4)
C3—C2—O1	132.5 (6)	C16—C15—H15A	119.9
C7—C2—O1	110.5 (6)	C14—C15—H15A	119.9
C9—N3—N2	113.1 (3)	C15—C16—C11	120.5 (3)
C2—C3—C4	123.1 (5)	C15—C16—H16A	119.8
C2—C3—H3B	118.5	C11—C16—H16A	119.8
C4—C3—H3B	118.5	C18—C17—H17A	109.5
O2—N4—O3	119.1 (4)	C18—C17—H17B	109.5
O2—N4—C14	121.2 (4)	H17A—C17—H17B	109.5
O3—N4—C14	119.7 (4)	C18—C17—H17C	109.5
C5—C4—C3	120.8 (5)	H17A—C17—H17C	109.5
C5—C4—H4A	119.6	H17B—C17—H17C	109.5
C3—C4—H4A	119.6	O4—C18—C17	108.2 (6)
C18—O4—P	122.8 (4)	O4—C18—H18A	110.1
C4—C5—C6	117.2 (4)	C17—C18—H18A	110.1
C4—C5—C8	124.1 (4)	O4—C18—H18B	110.1
C6—C5—C8	118.5 (4)	C17—C18—H18B	110.1
C20—O6—P	122.7 (3)	H18A—C18—H18B	108.4
C7—C6—C5	119.4 (5)	C20—C19—H19A	109.5
C7—C6—H6A	120.3	C20—C19—H19B	109.5
C5—C6—H6A	120.3	H19A—C19—H19B	109.5
C2—C7—C6	122.5 (5)	C20—C19—H19C	109.5
C2—C7—H7A	118.7	H19A—C19—H19C	109.5
C6—C7—H7A	118.7	H19B—C19—H19C	109.5
N1—C8—C5	112.2 (3)	O6—C20—C19	111.2 (5)
N1—C8—P	110.0 (3)	O6—C20—H20A	109.4
C5—C8—P	110.4 (3)	C19—C20—H20A	109.4
N1—C8—H8A	108.0	O6—C20—H20B	109.4
C5—C8—H8A	108.0	C19—C20—H20B	109.4
P—C8—H8A	108.0	H20A—C20—H20B	108.0
			
C1—O1—C2—C3	6.7 (9)	N2—N3—C9—N1	176.5 (4)
C1—O1—C2—C7	−176.9 (5)	N2—N3—C9—S	−0.5 (5)
C10—N2—N3—C9	1.4 (5)	C8—N1—C9—N3	0.6 (7)
C7—C2—C3—C4	2.2 (10)	C8—N1—C9—S	177.5 (3)
O1—C2—C3—C4	178.5 (6)	C10—S—C9—N3	−0.3 (3)
C2—C3—C4—C5	−0.9 (9)	C10—S—C9—N1	−177.5 (4)
O5—P—O4—C18	48.1 (5)	N3—N2—C10—C11	−177.9 (4)
O6—P—O4—C18	−78.7 (5)	N3—N2—C10—S	−1.6 (5)
C8—P—O4—C18	172.4 (5)	C9—S—C10—N2	1.0 (3)
C3—C4—C5—C6	0.3 (8)	C9—S—C10—C11	177.4 (4)
C3—C4—C5—C8	−174.3 (5)	N2—C10—C11—C12	−179.8 (4)
O5—P—O6—C20	−9.6 (5)	S—C10—C11—C12	4.3 (6)
O4—P—O6—C20	117.7 (5)	N2—C10—C11—C16	0.9 (6)
C8—P—O6—C20	−133.9 (4)	S—C10—C11—C16	−175.0 (3)
C4—C5—C6—C7	−1.1 (7)	C16—C11—C12—C13	3.5 (6)
C8—C5—C6—C7	173.8 (5)	C10—C11—C12—C13	−175.8 (4)
C3—C2—C7—C6	−3.0 (9)	C11—C12—C13—C14	−2.3 (7)
O1—C2—C7—C6	179.9 (5)	C12—C13—C14—N4	−179.2 (4)
C5—C6—C7—C2	2.5 (9)	C12—C13—C14—C15	−0.8 (7)
C9—N1—C8—C5	−136.1 (4)	O2—N4—C14—C13	178.9 (5)
C9—N1—C8—P	100.6 (4)	O3—N4—C14—C13	−3.6 (8)
C4—C5—C8—N1	−49.1 (6)	O2—N4—C14—C15	0.5 (8)
C6—C5—C8—N1	136.4 (4)	O3—N4—C14—C15	177.9 (5)
C4—C5—C8—P	74.1 (5)	C13—C14—C15—C16	2.5 (7)
C6—C5—C8—P	−100.5 (4)	N4—C14—C15—C16	−179.1 (5)
O5—P—C8—N1	53.6 (3)	C14—C15—C16—C11	−1.2 (7)
O6—P—C8—N1	178.1 (3)	C12—C11—C16—C15	−1.7 (7)
O4—P—C8—N1	−71.6 (3)	C10—C11—C16—C15	177.6 (4)
O5—P—C8—C5	−70.8 (3)	P—O4—C18—C17	−157.3 (5)
O6—P—C8—C5	53.7 (3)	P—O6—C20—C19	105.9 (5)
O4—P—C8—C5	164.0 (3)		

### Hydrogen-bond geometry (Å, °)

**Table:**

*D*—H···*A*	*D*—H	H···*A*	*D*···*A*	*D*—H···*A*
N1—H1A···O5^i^	0.86	1.94	2.782 (6)	165

Symmetry codes: (i) −*x*, −*y*, −*z*.
